# Mechanics of Wound Closure: Emerging Tape-Based Wound Closure Technology vs. Traditional Methods

**DOI:** 10.7759/cureus.827

**Published:** 2016-10-12

**Authors:** Kemal Levi, Kei Ichiryu, Pelin Kefel, Juergen Keller, Jon Grice, Ori Belson, Eric Storne, Bauback Safa

**Affiliations:** 1 R&D, Bio-X Technologies GmbH; 2 ZipLine Medical, Inc.; 3 Bio-X Technologies GmbH; 4 Plastic Surgery, Hand Surgery, Microsurgery, The Buncke Clinic

**Keywords:** wound closure, shear, mechanical strain, wound mechanics, dehiscence, scarring

## Abstract

To date, there is still a lack of understanding of how wound closure methods perform comparatively under daily bodily movement during the course of healing and how they affect the mechanics of healing. The present study is a first step in understanding and objectively quantifying the gap. The study provides both a new method of metrology for noninvasive evaluation of skin mechanics at the onset of wound healing and an emerging tape-based wound closure technology. The latter shows better performance with respect to commonly used staples and sutures, holding the wound intact and providing uniform mechanical support across the incision.

## Introduction

Karl Langer’s work [1] allowed surgeons to recognize that the stress state of skin has significant impact on wound closure and healing post surgery. Langer’s lines represent the directions of the skin’s maximum tension and are used as the preferred directions in which surgical incisions are made so that the tension across the wound is minimal post surgery [2]. Despite the widespread knowledge of the importance of protecting the wound from tension to prevent post-surgical complications (e.g. dehiscence [3] and fibrosis [4-5]), there is still a lack of understanding of how wound closure methods affect wound mechanics and perform while subject to physiologically relevant loading. In this study, we use biomechanical testing and digital image correlation (DIC), a noninvasive technique, to measure displacement and strains during loading of both non-biological and biological materials [6-8], to substantiate specific differences in how wound closure methods hold the skin tissue intact and impact the tensile strain distribution along the incision. We compare the efficacy of a tape-based wound closure method to subcuticular sutures and staples. Our analyses suggest that the tape-based technology distributes mechanical strains along the incision uniformly, preventing dehiscence, when the wound is subject to physiologically relevant loading. While staples have the highest holding strength, they fail to prevent dehiscence when skin is stretched and impart high shear at the incision front, increasing the likelihood of post-surgical scarring.

## Materials and methods

### Animals

Two adult Yorkshire pigs were used in accordance with the Institute for Laboratory Animal Research (ILAR) guidelines. Incision sites were identified where the skin was assessed to have an elasticity comparable to humans. The identified region was bordered between the lower aspect of the ribs and the hind limb and a few centimeters (cm) ventral to the spine and dorsal to the medial abdomen. Five incisions were made on the first pig, and eight on the second. A ~4 cm long incision was made at each site, resulting in ~5 mm of skin separation. The following devices were used for closure: 4-0 Vicryl suture (Ethicon, Somerville, NJ) in a subcuticular stitch, Appose ULC 35W staples (Covidien, Mansfield, MA) spaced approximately 8 mm apart, and Zip 8i Surgical Skin Closure Device (ZipLine Medical, Inc., Campbell, CA).

### Biomechanical studies

**Figure 1:**
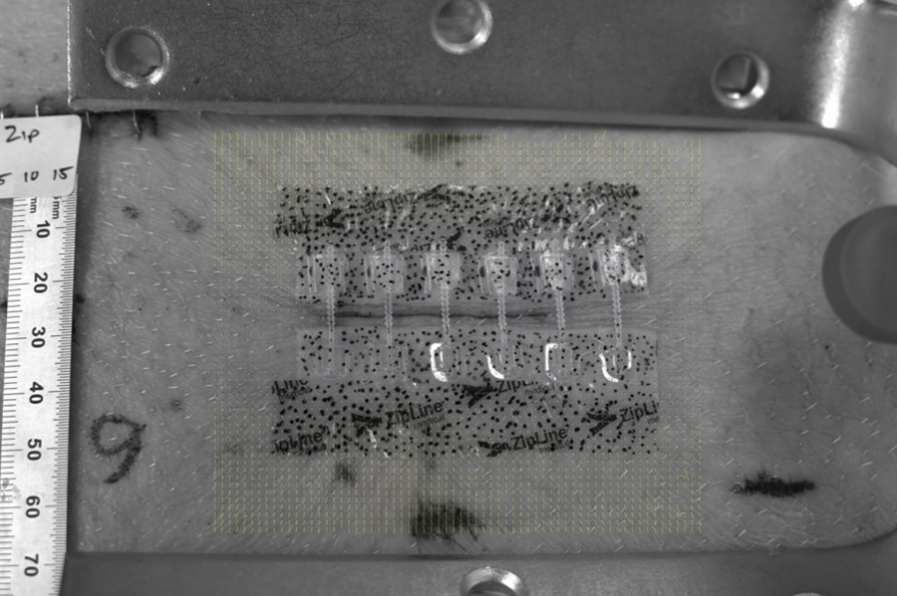
The apparatus used to stretch the skin on either side of the closed incision The yellow vectors indicate the direction of the displacement, measured with the DIC software, after stretching the skin.

DIC was used to evaluate the performance of each closure method in isolating the incision from physiologically relevant loading. The skin on either side of the closed incision was stretched to strains of five percent and 10% using a stretching apparatus as can be seen in Figure 1. High resolution images were captured after wound closure, apparatus application, and after loading. DIC software (Bio-X Technologies GmbH, Berlin, Germany), designed for tracking skin movement using the natural skin pattern, was used for image analysis.

**Figure 2:**
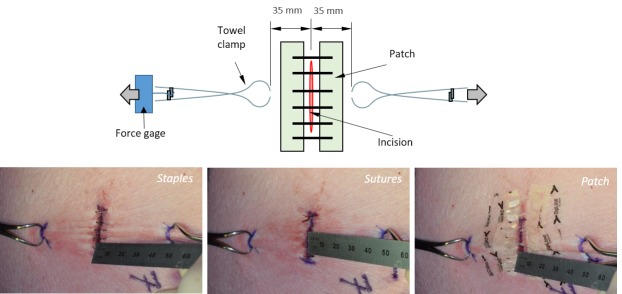
The holding strength and fracture mechanism for each closure method were evaluated using a uniaxial tension setup

The holding strength and fracture mechanism were evaluated with a wound tensioning setup as can be seen in Figure 2. After removal of the apparatus, towel clamps were applied to the skin ~35 mm away from each side of the incision. One clamp was held in place while the other was tensioned using a force gauge. The wound separation was visually assessed with a ruler. Force values, recorded at ~ 0.5 mm intervals of wound separation, were compared between the different wound closure methods. To control for mechanical property variations, half of the incision sites were closed with the tape-based device and the other half with sutures initially; staples were tested third to prevent them from disrupting the integrity of skin during the tensioning experiments. The pigs were euthanized at the conclusion of the study according to test facility protocols.

## Results

**Figure 3:**
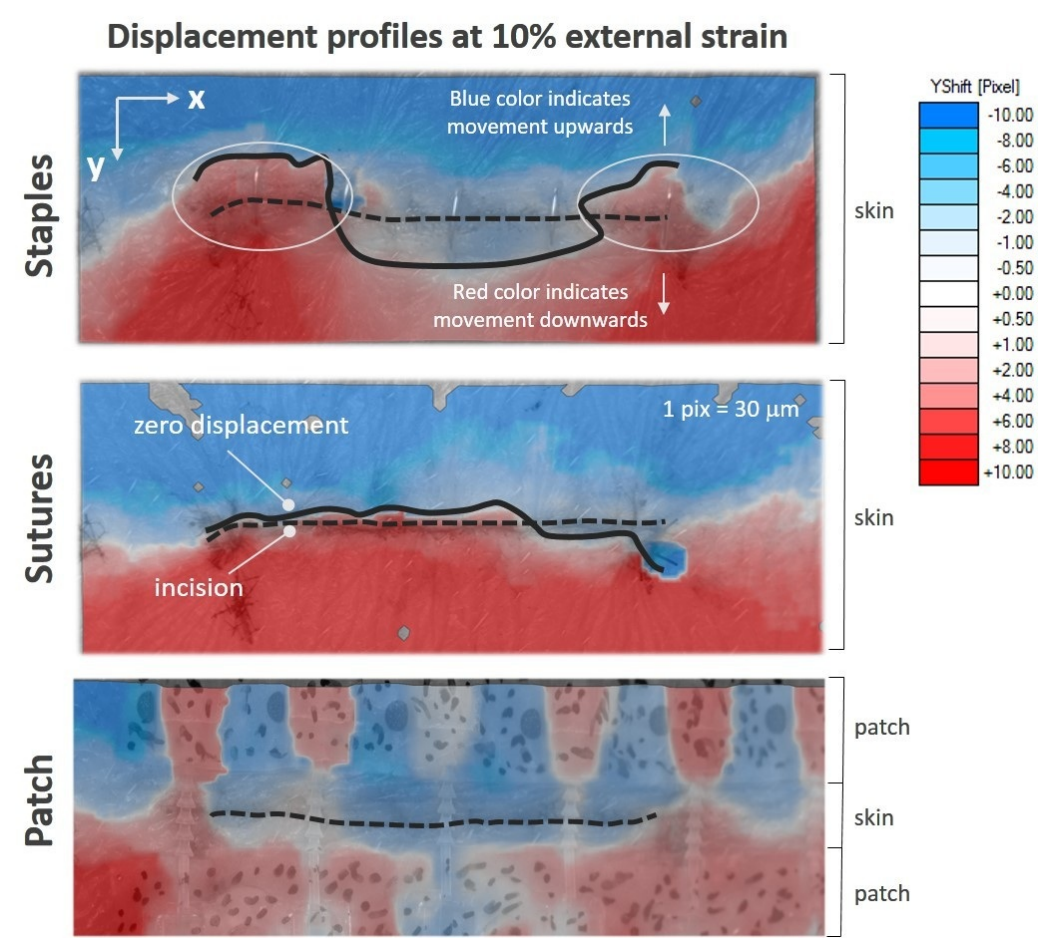
Displacement profiles at 10% external strain as a function of wound closure method In the case of staples, the incision front did not move in the same direction and did not separate uniformly in response to the stretch, as indicated by the zero displacement line and the color of the displacement vectors. Significant shear strains were observed at transition points (marked by white circles). Sutures were unable to hold the wound together, leading to wound dehiscence. Wound separation was uniform along the incision, as indicated by the sharp transition of color from red to blue across the incision. Compared to staples and sutures, the patch-based device showed better isolation from shear forces and held the wound intact, as indicated by the blue color across the incision.

For physiologically relevant loading, the DIC analysis showed that the tape-based device holds the wound better and isolates the wound from nonuniform strains across the incision as shown in Figure 3. In the case of staples, the incision front was placed under shear for all sites (n = 6). The incision front did not move in the same direction and did not separate uniformly during loading. This indicates the presence of significant shear strains at transition points, and that creates a high propensity for post-surgical scarring [9]. In the case of sutures, shear strains were lower compared to staples but higher compared to the tape-based device. In 40% of the sites, sutures were unable to hold the wound intact, leading to dehiscence. The tape-based device showed better isolation from shear across the incision compared to staples and sutures, holding the wound intact and keeping the strains uniform along the incision. This would lead to better wound healing and reduced scarring [10-11].

For hold strength, the tensioning experiments demonstrated that both staples and the tape-based device had a similar and significantly higher holding strength with respect to sutures. The holding strength for the tape-based device, sutures, and staples were 9.7 ± 3.1, 0.8 ± 0.5, and 12.5 ± 4.8 lbs, respectively. The difference between the tape-based device and staples was not statistically significant (p > 0.05); staples and the tape-based device had a significantly higher holding strength with respect to sutures (p < 0.01). Notably, while the forces required to dehisce the stapled wound were relatively high, the staples were observed to pinch on the healthy skin tissue around the staples, leading to a strong inflammatory reaction.

## Discussion

To our knowledge, this is the first study using biomechanical testing and digital image speckle correlation to demonstrate differences in how different wound closure methods perform under physiologically relevant loading and distribute tension. These preliminary studies may have important translational implications as the significance of isolating the wound from tension for improved wound closure and repair is increasingly recognized. In this regard, tape-based technologies are very promising, as they can be administered quickly and conform well to skin, providing uniform mechanical support across the incision over a much larger surface area than staples and sutures. Ongoing studies are being conducted to understand how variations in skin biomechanics for different age ranges, sex, and body regions impact wound tension and the efficacy of wound closure methods in accommodating this.

## Conclusions

We demonstrate how different wound closure methods impact the mechanics of the healing wound and the resulting performance using imaging and biomechanical methods. There were significant variations in their strain profiles during physiologically relevant loading. Emerging tape-based wound closure technology showed better performance with respect to traditional methods, holding the wound intact and protecting the incision from shear forces, which are known to cause increased scarring.
